# Early life migration and undernutrition among circular migrant children: An observational study in the brick kilns of Bihar, India

**DOI:** 10.7189/jogh.12.04008

**Published:** 2022-02-05

**Authors:** Reshma P Roshania, Rakesh Giri, Solveig A Cunningham, Melissa F Young, Amy Webb-Girard, Aritra Das, G S Mala, Sridhar Srikantiah, Tanmay Mahapatra, Usha Ramakrishnan

**Affiliations:** 1 Nutrition and Health Sciences, Laney Graduate School, Emory University, Atlanta Georgia, USA; 2 CARE India Solutions for Sustainable Development, Patna Bihar, India; 3 Hubert Department of Global Health, Rollins School of Public Health, Emory University, Atlanta, Georgia, USA

## Abstract

**Background:**

India holds the world's largest burden of chronic and acute child undernutrition. Poverty and systemic inequities are basic causes of undernutrition that also drive households to engage in circular migration for livelihood. Short-term, temporary movement of the whole family, including young children, is common; yet, the nutritional implications of recurrent movements beginning in early life has not been studied. We sought to estimate the association of repeat and early life migration with stunting and wasting outcomes among circular migrant children under three.

**Methods:**

Using a stratified cluster design, we conducted two waves of primary data collection among 2564 randomly selected circular migrant children under three years of age temporarily residing across 1156 brick kilns in Bihar, India. We conducted multilevel modeling to estimate the association of the number of migration episodes and age at first migration with stunting (<-2 standard deviations (SD) height-for-age z scores (HAZ)) and wasting (<-2 SD weight-for-height z-scores (WHZ)) and examined the determinants of nutrition status among migrant children, including diet, illness, food security and the health environment.

**Results:**

The overall prevalence of stunting was 51.6%. Among children who were either born during migration or first migrated before six months of age, those who experienced multiple episodes of migration were more likely to be stunted compared to those who migrated once (adjusted odds ratio (aOR) = 2.10; 95% confidence interval (CI) = 1.30-3.41). Children were over three times as likely to be wasted in the summer compared to the winter (aOR = 3.28; 95% CI = 2.68-4.01); in the summer, the overall prevalence of wasting was 38.8%. Public health access indicators such as interaction with frontline health workers at the destination was low (5.3%), whereas feeding indicators such as exclusive breastfeeding among 0-5 months was high (81.1%).

**Conclusions:**

Policy efforts should ensure continuity of social protection and welfare entitlements between home and destinations for circular migrant families, with an explicit focus on rural-to-rural movement.

India has 45.5 million children who are stunted, nearly one third of the global burden; of the 50 million children worldwide who are wasted, over one half live in India [1]. The basic causes of child stunting and wasting relate to the sociopolitical and economic contexts that limit or deny the realization of rights and access to essential resources among vulnerable populations, perpetuating poverty and inequity [2]. Indeed, countries that have experienced economic growth have also achieved national declines in stunting [3,4]. Although India has observed overall reductions in undernutrition during recent decades of growth, economic gains have been inequitable and disparities in child nutrition by household wealth have widened [5].

To cope with poverty and build wealth, many poor households engage in seasonal labor migration. The dominant form of labor migration undertaken by the chronically poor is circular in nature [6], defined as temporary, repetitive movement, followed by a return to the normal place of residence [7,8]. The most precarious streams of circular migration are undertaken by the very poor, less educated, and those from marginalized groups such as tribal and low-caste communities; the informality of employment can result in additional vulnerabilities in destination spaces with respect to working and living conditions. These streams often involve relatively short-distance (ie, intrastate or regional) movement of the entire family, including young children [7].

The majority of research on health and nutrition of children of internal migrants in India has focused on permanent, rural-to-urban migration streams [9]; these studies have found that migrant children are more likely to be undernourished and experience higher mortality compared to urban non-migrant children [10,11]. Studies examining nutrition among children who accompany their parents during seasonal migration have found low immunization [12], a high prevalence of undernutrition, and high morbidity [13,14]. However, there is a gap in the literature on the nutritional implications of repeated movement, characteristic of circular migration patterns, that considers migration beginning in early life, when nutritional insults can have a long-lasting impact.

The potential effects of circular migration on the nutrition status of accompanying children are theoretically multidirectional. Increased income may improve food security and dietary quality and intake, thereby improving child nutrition. Conversely, recurrent interruption of critical health services such as immunization, growth monitoring and maternal care due to cyclical movement, and unhygienic living conditions in many work sites may leave children vulnerable to illness and malnutrition.

We therefore conducted a study that allowed us to compare nutrition outcomes among children who have experienced multiple migrations, beginning from birth or early life, to those who have only experienced one migration. We focus our study in the state of Bihar, which has the highest prevalence of stunting among children under five in the country [15]; all 38 of Bihar’s districts classify as high (30% to <40%) or very high (≥40%) stunting [16]. Over one in five children under five years of age in the state are wasted [15], surpassing the World Health Organization (WHO) critical public health significance threshold of 15 percent [17]. Furthermore, Bihar ranks the lowest in the country in wealth [15], and has both the highest absolute and population percentage of temporary labor migration in the country [18]. We further focus our research in the country’s brick manufacturing industry, the second largest in the world after China [19], and growing in scale due to construction demands. Due to its location in the alluvial soil rich Gangetic plains, Bihar is a major brick producing state [20]. Brick making in India still largely utilizes traditional, manual processes of production, in some areas depending almost entirely on migrant labor [21]. Kilns are operational in the dry months (generally from October to June in Bihar), during which migrant families reside in rudimentary housing on the premises. Most kilns are located in rural and peri-urban areas; thus migrants who move for work in the brick industry classify as rural-to-rural migrants [22], a stream of labor migration underestimated in national statistics [7]. Due to the informality of the sector, estimates on the number of people working in brick kilns vary greatly; the International Labor Organization estimates there are over 140 000 kilns in India, each employing on average 50 to 100 laborers [23]. Women make up close to half of this workforce as a result of the industry’s practice of recruiting male-female pairs. This therefore results in the migration of children who accompany their parents and are consequently out of school.

The aims of this study are to: 1) examine the underlying and immediate determinants of nutrition status at brick kiln destination sites of circular migrant children; and 2) estimate the association of early life and repeat migration with stunting and wasting outcomes among circular migrant children.

## METHODS

This observational, cross-sectional study was part of an ongoing collaboration between Emory University and CARE India under the Bihar Technical Support Program funded by the Bill and Melinda Gates Foundation. Ethical approval was obtained from Emory University Institutional Review Board (IRB00090920) and Ashirwad Ethics Committee.

### Study design and sampling strategy

We employed a multi-stage sampling strategy, first stratifying by district, and then randomly selecting brick kiln clusters. Our sampling frame was obtained from the Department of Mines and Geology, which maintains a yearly updated list of all operational brick kilns in the state. The study was conducted in the 37 districts in Bihar that had listed operating kilns during the time of the study. We excluded kilns that did not have migrant families present (in other words, only employed local labor or unaccompanied male migrants), or that were closed either for the season or permanently. From this resulting sampling frame, based on a priori sample size calculations, we randomly selected 18 kilns per district using a random number generator in Microsoft Excel (Microsoft Inc, Seattle WA, USA). Within each selected kiln, a census was conducted to identify circular migrant families meeting the inclusion criteria for the study: 1) self-identification as a circular migrant household, defined as living away from their home block (sub-district) for employment purposes for a total of at least 60 days in the previous year, with at least one return home during that year; and 2) presence of at least one child under three years of age at the kiln. Using a random number table, three eligible families per kiln were selected, one with a child in the zero to eleven month age group and two with a child in the 12 to 35-month age group. Children zero to eleven months of age were oversampled to ensure an adequate sample size for performing subgroup analyses. Verbal informed consent was obtained from the mother of each selected child before the interview and anthropometric measurement. No incentives were provided to the participants. We collected two waves of cross-sectional data in June 2018 (summer) and January 2019 (winter) to examine seasonality. The summer wave of data was collected towards the end of the migration season, and therefore reflected a longer average length of time since arrival to the kiln of enumeration compared to the winter wave of data collection, which occurred approximately two months into the migration season.

### Data collection

After orienting the kiln owner or manager to the purposes of our study, trained CARE India staff administered a household survey to the selected child’s mother and collected anthropometric data on all selected children. Enumerators recorded anthropometric and survey data digitally on Android tablets using a custom interface developed with SurveyCTO software (Dobility Inc, Cambridge MA, USA), which included in-built logic checks and data validation. Supervisory staff additionally conducted back-checks with a random subsample of respondents to ensure data quality.

### Variables

Our key exposures of interest were child age at first migration (in months) and number of migration episodes since the index child’s birth (including the migration at the time of survey), derived from detailed questions on the family’s migration history. Primary outcomes were height-for-age z-scores (HAZ) and weight-for-height (WHZ) z-scores, both calculated using WHO reference data [24]. HAZ and WHZ scores of less than negative two standard deviations (SD) classify as stunting and wasting, respectively. Additional variables of interest included the basic, underlying and immediate determinants of nutrition: basic determinants include wealth, level of education completed by mother of selected child, parity, state of origin, and caste. Scheduled Castes, or Dalits, are marginalized Hindu communities that are outside of the traditional caste hierarchy. Scheduled Tribes are marginalized communities due to geographic isolation. Other Backward Classes are marginalized communities that are economically, socially or educationally disadvantaged and do not fall into the Scheduled Caste or Scheduled Tribe classifications. Underlying determinants include sanitation on the kiln, household food insecurity, and health care access (full immunization, and interaction with front-line health workers since arrival to the kiln). Front-line health workers include Anganwadi Workers, Accredited Social Health Activists, and Auxiliary Nurse Midwives; these cadres of community-based health workers deliver essential health and nutrition services such as immunization, food supplements, institutional delivery promotion, and education about recommended feeding practices. Immediate determinants of nutrition include illness in the previous one month (acute respiratory infection (ARI) and diarrhea), and age appropriate feeding. Relative wealth index was derived by conducting principal component analysis of household asset ownership data and creating cohort-based quintiles based on the resulting factor scores [25]. Household food insecurity was measured using the Food and Agriculture Organization Food Insecurity Experience Scale (FIES) [26], with reference to the previous one year and was dichotomized into any vs no food insecurity. ARI was presumed if any one of the following symptoms were reported by the mother: cough with fast breathing, difficulty in breathing, chest indrawing, or wheezing/grunting. Age appropriate feeding was a dichotomous variable constructed based on age-specific WHO infant and young child feeding indicators [27]. For children zero to five months, age appropriate feeding was defined as exclusive breastfeeding. Exclusive breastfeeding was only included in the January 2019 wave of data collection; as a result, children zero to five months from the June 2018 wave were excluded from analyses using the age appropriate feeding indicator (n = 111). For children 6 to 23 months, age appropriate feeding was defined as minimum acceptable diet using a dietary quality screening tool developed by CARE India. For children 24 to 35 months, age appropriate feeding was defined as dietary diversity (greater than or equal to four food groups) using 24-hour recall data [28].

### Statistical analysis

We first conducted descriptive analyses of sociodemographic characteristics and migration exposures of our sample, followed by bivariate associations between the determinants of nutrition status and season, using Rao-Scott χ^2^ tests. We estimated the unadjusted prevalence of stunting and wasting by wave, sex, and age; age was categorized into nutritionally relevant groupings based on infant and young feeding recommendations [27].

To examine the adjusted association of migration exposures with nutrition status, we ran multiple variable logistic regression models with dichotomous stunting and wasting as separate outcomes. We tested statistical interaction between child age at first migration (categorized into birth during migration or first migration at less than six months, and first migration at greater than or equal to six months) and number of migration episodes (categorized into one, two and greater than or equal to three episodes) for both nutrition outcomes. For each outcome, we ran models controlling for age in months, sex, state of origin (categorized into Bihar, Jharkhand, and Other states), caste, parity, mother’s education, wealth quintile and season.

Collinearity of predictor variables were assessed for all models. All analyses accounted for the stratified cluster survey design. We did not include survey weights as the true population distribution of migrant children is unknown. Alpha was set at 0.05, and analyses were conducted in SAS (SAS Institute Inc, Cary NC, USA), version 9.4. Figures were generated using R (R Foundation for Statistical Computing, Vienna, AUT), version 4.0.2.

## RESULTS

The study had a final sample size of 2564 children 0 to 35 months of age from both waves of data (1094 from the June 2018 wave and 1470 from the January 2019 wave) after excluding observations with missing or implausible outcome values (n = 178). 519 and 637 brick kilns were surveyed in the first and second waves, respectively. Reasons for not surveying a selected eligible kiln included refusal by the kiln owner or manager to enter the premises, or inability to locate the kiln.

### Population characteristics

**Figure 1:**
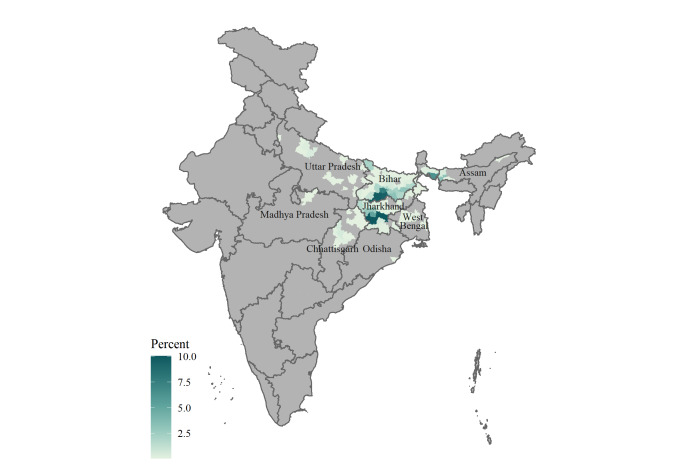
District origin distribution of circular migrant families residing at study site brick kilns in Bihar, June 2018 and January 2019.

The majority of migrants were from Bihar (53.4%), followed by the neighboring state of Jharkhand (33.2%). Figure 1 displays the distribution of migrant origins by district, showing the high presence of migrants on selected kilns from Gaya, Nawada and Nalanda districts in Bihar, Ranchi and Gumla districts in Jharkhand and Cooch Behar district in West Bengal represented in our study.

**Table 1** demonstrates differences in sociodemographic characteristics by state of origin. Interstate migrants had greater representation in higher wealth quintiles compared to those who migrated within Bihar. On average, intrastate migrants had larger households, migrated with a greater number of household members and have engaged in circular migration for a greater number of years, compared to migrants from other states. Among respondents who migrated for work for more than one year (n = 2143), 77 percent worked only in the brick kiln setting during previous migrations, followed by six percent of circular migrants who reported also migrating for work in agriculture (data not shown).

**Table 1:** Sociodemographic characteristics of circular migrant families in Bihar by state of origin, June 2018 and January 2019

	**Bihar**	**Jharkhand**	**Other**	**Total**
**All**, n (%)	1370 (53.4)	851 (33.2)	343 (13.4)	2564 (100.0)
Child age category* (months), n (%)				
0 to 5	239 (17.4)	86 (10.1)	50 (14.6)	375 (14.6)
6 to 11	231 (16.9)	160 (18.8)	63 (18.4)	454 (17.7)
12 to 23	616 (45.0)	389 (45.7)	126 (36.7)	1131 (44.1)
24 to 35	284 (20.7)	216 (25.4)	104 (30.3)	604 (23.6)
**Child sex**, n (%)				
Male	686 (50.1)	426 (50.1)	187 (54.5)	1299 (50.7)
Female	684 (49.9)	425 (49.9)	156 (45.5)	1265 (49.3)
**Caste**, n (%)				
Scheduled Caste	944 (68.9)	189 (22.2)	26 (7.6)	1159 (45.2)
Scheduled Tribe	93 (6.8)	533 (62.6)	22 (6.4)	648 (25.3)
Other Backward Class	321 (23.4)	119 (14)	160 (46.6)	600 (23.4)
General Caste	12 (0.9)	10 (1.2)	135 (39.4)	157 (6.1)
**Religion**, n (%):				
Hindu	1358 (99.1)	788 (92.6)	101 (29.4)	2247 (87.6)
Muslim	12 (0.9)	21 (2.5)	241 (70.3)	274 (10.7)
Other	0 (0.0)	42 (4.9)	1 (0.3)	43 (1.7)
**Mother’s age **(years; mean, SD) (n = 2268)	25.8 (5.3)	24.3 (4.7)	24.2 (5.3)	25.0 (4.9)
**Mother’s age at marriage **(years; mean, SD) (n = 2550)	15.9 (3.0)	16.7 (3.2)	15.8 (3.6)	16.4 (3.3)
**Parity of mother **(mean, SD)	3.4 (1.9)	2.6 (1.6)	2.5 (1.5)	2.6 (1.5)
**Mother’s education**, n (%)				
No formal education	1308 (95.5)	640 (75.2)	229 (66.8)	2177 (84.9)
Up to 8th standard	52 (3.8)	131 (15.4)	90 (26.2)	273 (10.6)
Above 8th standard	10 (0.7)	80 (9.4)	24 (7.0)	114 (4.4)
**Father’s education**, n (%) (n = 2430)				
No formal education	1128 (85.6)	439 (56.4)	229 (68.6)	1796 (73.9)
Up to 8th standard	158 (12.0)	201 (25.8)	96 (28.7)	455 (18.7)
Above 8th standard	32 (2.4)	138 (17.7)	9 (2.7)	179 (7.4)
**Primary occupation of father**, n (%) (n = 1458)				
Agriculture/agricultural labor	371 (44.9)	229 (65.1)	106 (38.0)	706 (48.4)
Non-agricultural labor	369 (44.6)	84 (23.9)	123 (44.1)	576 (39.5)
Unemployed	63 (7.6)	25 (7.1)	23 (8.2)	111 (7.6)
Other	24 (2.9)	14 (4.0)	27 (9.7)	65 (4.5)
**Wealth quintile**, n (%)				
Lowest	402 (29.3)	86 (10.1)	29 (8.5)	517 (20.2)
Second	349 (25.5)	121 (14.2)	38 (11.1)	508 (19.8)
Middle	303 (22.1)	153 (18.0)	57 (16.6)	513 (20.0)
Fourth	223 (16.3)	196 (23.0)	94 (27.4)	513 (20.0)
Highest	93 (6.8)	295 (34.7)	125 (36.4)	513 (20.0)
**Household size† at origin**, mean (SD)	6.4 (2.6)	6.5 (2.9)	5.7 (2.3)	6.3 (2.7)
**Household size at kiln**, mean (SD)	5.3 (1.8)	4.2 (1.4)	4.5 (1.5)	4.8 (1.7)
**Number of years migrating‡**, mean (SD)	6.1 (4.6)	5.2 (4.5)	4.3 (3.7)	5.6 (4.5)

SD – standard deviation

*Child age at time of survey

†The definition of a household is a group of people who eat meals from the same kitchen.

‡The number of years the respondent reported migrating for any work.

### Migration exposures

About half of the children (45.5%) sampled in our study experienced more than one migration episode; 12.6% experienced three or more migration cycles. Over half of children sampled were early life migrants; that is, they were either born during migration (16.4%) or first experienced migration between zero and five months of age (39.1%). Socioeconomic characteristics by age at first migration of the index child are presented in Table S1 in the **Online Supplementary Document****.**

Reflecting the seasonality of migration, the average number of months spent at the destination kiln during the current migration episode was 6.0 (SD = 2.0) months in the summer wave of data collection, and 2.8 (SD = 1.5) months in the winter wave of data collection.

### Underlying and immediate determinants of nutrition status at the destination

Overall, access to health services in the study sample was low; 42.5% of children 12 to 23 months were fully immunized, and 5.3% of respondents reported interactions with front-line health workers at the migration destination. 37.2% of households experienced food insecurity in the previous year. Most children were in environments without any sanitation facilities; 84.6% of respondents reported open defecation at the kiln. Only 18.3% of children had a diet that met age appropriate feeding guidelines; this was primarily driven by feeding practices for children over six months of age: 81.1% of children zero to five months were exclusively breastfed; 15.3% of children 6 to 23 months received a minimum acceptable diet, and 4.3% of children 24 to 35 months consumed greater than or equal to four food groups in the previous 24 hours.

**Figure 2:**
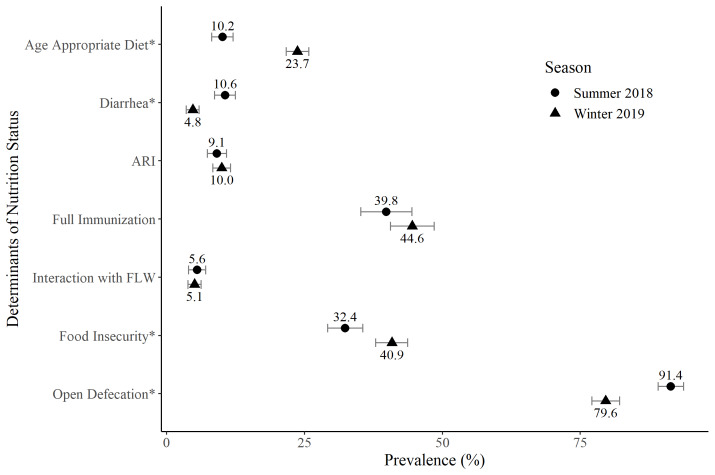
Prevalence of the determinants of nutrition status by season among circular migrant children 0-35 months old, June 2018 and January 2019, Bihar. The underlying determinants of nutrition status are: sanitation access (measured by open defecation), food insecurity, and health care access (interaction with front-line health workers (FLW), and full immunization). The immediate determinants of nutrition status are: illness (acute respiratory infection (ARI) and diarrhea), and age appropriate diet. *Statistically significantly different at the 0.05 level. Error bars represent 95% confidence intervals. n = 2564, except for full immunization (measured among children 12-23 months of age, n = 1064), food insecurity (n = 2560) and age appropriate diet (n = 2453).

We observed significant differences in the determinants of nutrition status by season. In the summer, there was a lower proportion of children who met age-appropriate feeding recommendations, a higher prevalence of reported open defecation, and a higher prevalence of diarrhea compared to the winter (**Figure 2**). Significantly higher food insecurity was reported in the winter compared to the summer. There were no differences by season in ARI prevalence, full immunization or contact with front-line health workers.

### Stunting and wasting

**Figure 3:**
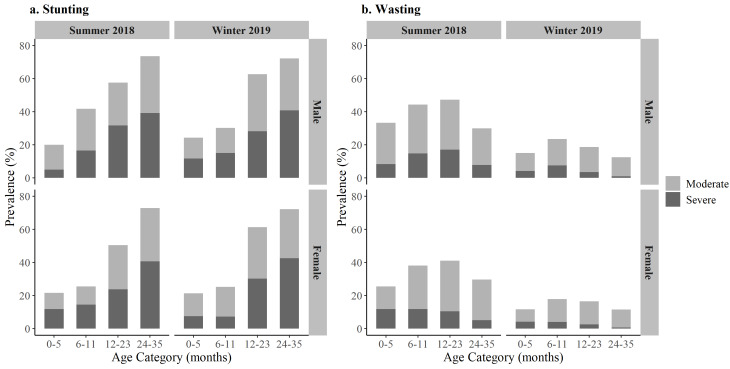
Stunting (panel a) and wasting (panel b) prevalence among circular migrant children 0-35 months old by age category, sex, and season, June 2018 and January 2019, Bihar. Severe Stunting/Wasting:<-3 standard deviations (SD) height-for-age z-scores (HAZ) / weight-for-height z-scores (WHZ); Moderate Stunting/Wasting: -3 to < -2 SD.

The overall unadjusted prevalence of stunting was 51.6%; among children who were stunted, half were severely stunted (25.9%). Stunting prevalence significantly increased with age category (*P* < 0.0001) (**Figure 3**, Panel a). Males were more likely to be stunted compared to females (*P* = 0.021); there were no differences in stunting prevalence by season of data collection.

Overall, wasting and severe wasting among the sampled children was 25.7% and 6.7%, respectively. The highest prevalence of wasting was in the six to eleven and 12 to 23 months age categories for males and females, and in both seasons. (**Figure 3**, Panel b). Males were more likely to be wasted compared to females (*P* = 0.025). There were significant differences by season in wasting prevalence: 38.8% of children overall were wasted in the summer compared to 16.0% during the winter (*P* < 0.0001).

We found evidence of statistically significant interaction between child age at first migration and number of migration episodes. Among children who first migrated early in life (who were either born during a migration episode or who first migrated within the first six months of life), those who migrated multiple times had higher odds of stunting compared to children who migrated once. (**Table 2**), adjusting for age, sex, season and pre-migration characteristics. Among children who first migrated after six months of age, there is no association of number of migrations with stunting.

**Table 2:** Adjusted estimates of stunting among circular migrant children 0-35 months old, June 2018 and January 2019, Bihar

	OR (95% CI)
**Interaction of number of migration episode**s* **and child age at first migration**	
2^nd^ vs 1^st^ migration, age at first migration <6 months	1.60 (1.17-2.19)
≥3^rd^ vs 1^st^ migration, age at first migration <6 months	2.10 (1.30-3.41)
2^nd^ vs 1^st^ migration, age at first migration ≥6 months	0.93 (0.69-1.26)
≥3^rd^ vs 1^st^ migration, age at first migration ≥6 months	1.01 (0.61-1.70)
**Child age** (months)†	1.08 (1.06-1.10)
**Child sex**	
Female	0.80 (0.68-0.96)
Male	Reference
**Origin**	
Bihar	Reference
Jharkhand	0.77 (0.60-0.99)
Other	0.58 (0.41-0.82)
**Caste**	
Scheduled Caste	1.64 (1.30-2.06)
Scheduled Tribe	1.27 (0.95-1.68)
General Caste	1.09 (0.69-1.73)
Other Backward Class	Reference
**Parity of mother**	
≥4	1.05 (0.87-1.26)
≤3	Reference
**Mother’s education**	
Any education	0.86 (0.67-1.11)
No education	Reference
**Wealth quintile**	
Lowest	1.19 (0.87-1.63)
Second	1.10 (0.81-1.49)
Middle	0.99 (0.75-1.32)
Fourth	0.91 (0.69-1.20)
Highest	Reference
**Season:**	
Summer 2018	0.83 (0.69-0.99)
Winter 2019	Reference

OR – odds ratio, CI – confidence interval

*The number of times child migrated since birth, including the migration at time of survey.

†Child age at time of survey.

We did not find any evidence of interaction between child age at first migration and number of migration episodes for wasting. Neither age at first migration nor number of migration episodes were associated with the odds of wasting (**Table 3**). The strongest driver of wasting was the summer season; children from the summer wave of data were over three times as likely to be wasted compared to the winter.

**Table 3:** Adjusted estimates of wasting among circular migrant children 0-35 months old, June 2018 and January 2019, Bihar

	OR (95% CI)
Number of migration episodes*	
1	Reference
2	0.95 (0.74-1.22)
≥3	0.73 (0.49-1.09)
**Child age at first migration (months)**	
<6	0.99 (0.77-1.28)
≥6	Reference
**Child age** (months)†	0.99 (0.98-1.01)
**Child sex**	
Female	0.81 (0.68-0.97)
Male	Reference
**Origin**	
Bihar	Reference
Jharkhand	1.46 (1.13-1.88)
Other	1.17 (0.79-1.76)
**Caste**	
Scheduled Caste	1.24 (0.95-1.61)
Scheduled Tribe	1.14 (0.85-1.54)
General Caste	1.48 (0.91-2.42)
Other Backward Class	Reference
**Parity of mother**	
≥4	1.18 (0.96-1.46)
≤3	Reference
**Mother’s education**	
Any education	1.01 (0.76-1.34)
No education	Reference
**Wealth quintile**	
Lowest	1.12 (0.80-1.55)
Second	1.28 (0.92-1.76)
Middle	1.32 (0.97-1.80)
Fourth	1.11 (0.82-1.49)
Highest	Reference
**Season**	
Summer 2018	3.28 (2.68-4.01)
Winter 2019	Reference

OR – odds ratio, CI – confidence interval

*The number of times child migrated since birth, including the migration at time of survey.

†Child age at time of survey.

## DISCUSSION

Our study found that among circular migrant children who were born during migration or first migrated in the early months of life, experiencing multiple episodes of migration was associated with a greater likelihood of stunting compared to children who were experiencing their first migration at the time of the study, suggesting a cumulative association of migration with stunting. These results can potentially be explained by shifts in the underlying and immediate conditions that determine nutrition status as outlined in the UNICEF conceptual framework of the determinants of undernutrition [2].

We found only 42% of circular migrant children 12 to 23 months of age received full immunization, an important indicator of regular access to the health system, compared to 62% of children nationally in the same age group who are fully immunized [15]. Families engaged in circular migration live away from their residence for much of the year, often during pregnancy and delivery, disrupted from their home sources of health care and information. At the destination, circular migrants can be disconnected from health services due to long work hours, unfamiliarity with the local health system, language barriers, and discrimination against migrants.

Age appropriate feeding substantially varied by age. For example, over 80 percent of infants under six months were exclusively breastfed, whereas only 15 percent of children 6 to 23 months received a diet considered acceptable in terms of frequency of meals and diversity of foods. This gap may be due to the financial and time challenges of meeting the complementary feeding guidelines of frequent, diverse meals. Notably, age appropriate feeding in both age groups were higher than in national estimates: 55 percent of infants are exclusively breastfed and under ten percent of children 6 to 23 months of age receive a minimum acceptable diet nationally [15]. This pattern suggests that some migrant families may have a better ability to implement recommended infant feeding practices compared to the overall population. One study conducted in informal settlements in Mumbai comprising of mainly migrants found that social support and self-efficacy for decision making are important factors determining mothers’ feeding behaviors [29]; it is possible that for some women, these constructs can improve during migration, as they are removed from traditional, sometimes oppressive, family structures. Additionally, migration can influence children’s diets (positively or negatively) through increased household income as well as exposure to new health and nutrition practices [30].

We found a high prevalence of wasting among child migrants in the summer season, which coincides with the end of the brick production season. Literature on the effects of seasonality on nutrition status in the region finds wasting is more prevalent during the rainy season compared to the dry season [31,32]; the proposed pathways include higher incidence of infectious disease and pre-harvest food unavailability during the monsoon. In the context of seasonal migration for work in the brick industry, migrants often return home before the onset of monsoon as traditional brick production does not operate during the rainy months; however, the same pathways are relevant for this population. Indeed, we observed higher diarrhea prevalence in the summer wave of data collection. Diarrheal pathogen counts increase in the warmer summer months compared to the winter [33], as do densities of flies, vectors of enteric disease [34]; these factors compounded with the widespread absence of sanitation facilities for migrants on kilns increase the risk of diarrhea in the summer. We also observed lower age appropriate feeding in the summer compared to the winter, potentially due to less availability of diverse foods and higher food prices in the summer compared to the winter. Since weight is sensitive to short-term changes in food intake and illness, the accumulative effects of lack of sanitation on the kiln and changes in the food environment over the six to eight-month migration season may contribute to a higher prevalence of wasting towards the end of the migration cycle.

Migration experts have long articulated recommendations for the explicit inclusion of internal migrants in the country’s social protection and nutrition policy framework, beginning with accurate enumeration of temporary migrants and women who migrate for work. Additionally, recommendations include portability of entitlements to subsidized food rations through the Public Distribution System, expanded coverage of the Integrated Child Development Services front-line health workers to include women and children who move, and implementation of existing legislation for safe housing on construction sites inclusive of adequate sanitation facilities [7,22,35]. These recommendations have largely focused on addressing the systematic exclusion of migrants from urban spaces. Our findings illustrate the gaps in entitlements and delivery of services among families who engage in rural-to-rural migration; policy efforts must also consider the enumeration and needs of migrants in rural destination environments, which often are less regulated and more disconnected from the public health system.

There are some limitations to this study. Due to the nature of the cross-sectional design of the study, we are unable to conclude that early life and repeat migration causes poor height-for-age outcomes; additionally, we cannot estimate the extent to which growth trajectories falter or accelerate over the course of a migration episode, or the influence on growth trajectories of the determinants of nutrition status, such as illness and food security, which also seasonally fluctuate. Our results could be upward biased by residual confounding from covariates we did not include in our analysis, for example maternal nutrition. Lastly, these findings are not generalizable beyond the population studied of circular migrant children who temporarily reside at mostly rural brick kilns in Bihar. Brick kilns present a unique nutritional risk environment with respect to pollution and sanitation; conducting similar research in alternative contexts of family circular migration, such as agriculture, will be useful.

There are several strengths to this research. We had a large, state-wide sample with a comprehensive sampling frame of all legally operating kilns in the state. By collecting detailed migration history, we were able to explore the association of nutrition with early life and repeat migration, characteristic of circular patterns of temporary movement; this is the first study to our knowledge to examine the association of these patterns with nutrition. Additionally, we conducted our research in Bihar, which experiences the greatest circular migration in the country. Given the high occurrence of family movement, and the poor coverage of health services in general, multi-sectoral and coordinated targeting by government health, rural development and labor ministries is a necessary strategy to improve the health and nutrition status of women and children in the state overall. Our work contributes to the migration and nutrition literature by examining circular, rural-to-rural, and family migration – streams that have been less addressed in existing research.

In conclusion, circular child migrants who have migrated multiple times beginning early in life are especially vulnerable to chronic undernutrition compared to children who have only experienced one migration. An alarmingly high percentage of circular child migrants temporarily residing on brick kilns are acutely malnourished relative to the overall population of Indian children, and are therefore are at an increased risk of mortality, especially as the migration season progresses into summer. However, it is important to recognize that engaging in migration may also be advantageous for children in some nutritional aspects such as dietary diversity and feeding practices. Further research is required to understand the complex and multidimensional pathways of circular migration and child nutrition. Policy efforts should emphasize accurate enumeration of circular migrants, regulate employers to ensure safe living and working conditions and fair pay, and ensure continuity of food security and health entitlements between home and destinations for households that engage in circular migration.

## Additional material


Online Supplementary Document

## References

[1] Development Initiatives. 2018 Global Nutrition Report: Shining a light to spur action on nutrition. Bristol, UK: Development Initiatives, 2018.

[2] UNICEF. UNICEF's approach to scaling up nutrition for mothers and their children. New York: Programme Division, United Nations Children's Fund, 2015.

[3] Haddad L Alderman H Appleton S Song L Yohannes Y Reducing child malnutrition: How far does income growth take us? World Bank Econ Rev. 2003;17:107-31. 10.1093/wber/lhg012

[4] Headey DD Developmental drivers of nutritional change: A cross-country analysis. World Dev. 2013;42:76-88. 10.1016/j.worlddev.2012.07.002

[5] Subramanyam MA Kawachi I Berkman LF Subramanian SV Socioeconomic inequalities in childhood undernutrition in India: Analyzing trends between 1992 and 2005. PLoS One. 2010;5:e11392. 10.1371/journal.pone.0011392 20617192 PMC2894973

[6] Bird K, Deshingkar P. Circular migration in India: Policy brief no. 4. London: Overseas Development Institute, 2009.

[7] Deshingkar P, Farrington J. A framework for understanding circular migration. In: Deshingkar P, Farrington J, editors. Circular Migration and Multilocational Livelihood Strategies in Rural India. New Delhi: Oxford University Press; 2009. p. 01-36.

[8] Zelinsky W The hypothesis of the mobility transition. Geogr Rev. 1971;61:219-49. 10.2307/213996 PMC7269166 32494088

[9] Kusuma YS Babu BV Migration and health: A systematic review on health and health care of internal migrants in India. Int J Health Plann Manage. 2018;33:775-93. 10.1002/hpm.2570 30074640

[10] Prusty RK Keshri K Differentials in child nutrition and immunization among migrants and non-migrants in urban India. Int J Migr Health Soc Care. 2015;11:194-205. 10.1108/IJMHSC-02-2014-0006

[11] Stephenson R Matthews Z McDonald JW The impact of rural-urban migration on under-two mortality in India. J Biosoc Sci. 2003;35:15-31. 10.1017/S0021932003000154 12537153

[12] Pakhare AP Pawar R Lokhande GS Datta SS Does seasonal migration for sugarcane harvesting influence routine immunization coverage? A cross-sectional study from rural Maharashtra. Indian J Public Health. 2014;58:116. 10.4103/0019-557X.132288 24820986

[13] Biswas T Mandal P Biswas S Assessment of health nutrition and immunisation status amongst under-5 children in migratory brick klin population of periurban Kolkata India. Sudan J Public Health. 2011;6:7-13.

[14] Mali KH Sawardekar P Anjenaya S Assessment of malnutrition in 1-5 years old children of brick-kiln workers in rural part near municipal area. New Indian Journal of Pediatrics. 2017;6:225-9.

[15] International Institute of Population Sciences. National Family Health Survey (NFHS-4), 2015–16: India. Mumbai: IIPS, 2017.

[16] Menon P Headey D Avula R Nguyen PH Understanding the geographical burden of stunting in India: A regression-decomposition analysis of district-level data from 2015-16. Matern Child Nutr. 2018;14:e12620. 10.1111/mcn.12620 29797455 PMC6175441

[17] World Health Organization. Nutrition Landscape Information System (‎NLIS)‎ country profile indicators: Interpretation guide. Geneva: World Health Organization, 2010.

[18] Keshri K, Bhagat RB. Temporary labour migration in India: Regional patterns, characteristics and associated factors. In: Bhagat RB, Roy AK, Sahoo H, editors. Migration and Urban Transition in India: A Development Perspective. First ed: Routledge India; 2020.

[19] Kamyotra JS. Brick kilns in India. Centre for Science and Environment Anil Agarwal Dialogue 2015: Poor in climate change; 2015 March 11-12; New Delhi.

[20] Development Alternatives. Status of brick sector in the state of Bihar: A baseline study. New Delhi: Development Alternatives, 2012.

[21] Singh DP Women workers in the brick kiln industry in Haryana, India. Indian J Gend Stud. 2005;12:83-97. 10.1177/097152150401200104

[22] Working Group on Migration. Report of the Working Group on Migration. New Delhi: Ministry of Housing and Urban Poverty Alleviation, Government of India, 2017.

[23] Mitra D, Valette D. Brick by brick: Unveiling the full picture of South Asia’s brick kiln industry and building the blocks for change. Geneva: Fundamental Principles and Rights at Work Branch, International Labour Organization, 2017.

[24] WHO Multicentre Growth Reference Study Group WHO Child Growth Standards based on length/height, weight and age. Acta Paediatr Suppl. 2006;450:76-85. 16817681 10.1111/j.1651-2227.2006.tb02378.x

[25] Vyas S Kumaranayake L Constructing socio-economic status indices: How to use principal components analysis. Health Policy Plan. 2006;21:459-68. 10.1093/heapol/czl029 17030551

[26] Cafiero C Viviani S Nord M Food security measurement in a global context: The food insecurity experience scale. Measurement. 2018;116:146-52. 10.1016/j.measurement.2017.10.065

[27] World Health Organization. Indicators for assessing infant and young child feeding practices part 2: Measurement. Geneva: World Health Organization; 2010.

[28] Gibson RS. Principles of Nutritional Assessment. Second ed. New York: Oxford University Press; 2005.

[29] Athavale P Hoeft K Dalal RM Bondre AP Mukherjee P Sokal-Gutierrez K A qualitative assessment of barriers and facilitators to implementing recommended infant nutrition practices in Mumbai, India. J Health Popul Nutr. 2020;39:7. 10.1186/s41043-020-00215-w 32718334 PMC7385866

[30] Zezza A Carletto C Davis B Winters P Assessing the impact of migration on food and nutrition security. Food Policy. 2011;36:1-6. 10.1016/j.foodpol.2010.11.005

[31] Brown KH Black RE Becker S Seasonal changes in nutritional status and the prevalence of malnutrition in a longitudinal study of young children in rural Bangladesh. Am J Clin Nutr. 1982;36:303-13. 10.1093/ajcn/36.2.294 6808822

[32] Hillbruner C Egan R Seasonality, household food security, and nutritional status in Dinajpur, Bangladesh. Food Nutr Bull. 2008;29:221-31. 10.1177/156482650802900308 18947035

[33] Saha S Halder M Mookerjee S Palit A Seasonal influence, enteropathogenic microbial load and diarrhoeal enigma in the Gangetic Delta, India: Present scenario and health implications. J Infect Public Health. 2019;12:540-8. 10.1016/j.jiph.2019.01.066 30792073

[34] Chavasse DC Shier RP Murphy OA Huttly SRA Cousens SN Akhtar T Impact of fly control on childhood diarrhoea in Pakistan: Community-randomised trial. Lancet. 1999;353:22-5. 10.1016/S0140-6736(98)03366-2 10023946

[35] Behera MR Health and policy environment of internal labour migrants in India: A literature review and future direction. International Journal of Current Research and Review. 2018;10:1-7. 10.31782/IJCRR.2018.10191

