# Supplementary material for: Early life migration and undernutrition among circular migrant children: An observational study in the brick kilns of Bihar, India
Source: J Glob Health. 2022 Feb 5;12:04008. doi: 10.7189/jogh.12.04008 (PMC8818295; doi:10.7189/jogh.12.04008)
Supplement: Online Supplementary Document [file jogh-12-04008-s001.pdf]

Table S1. Sociodemographic Characteristics of Circular Migrant Households by Child Age at First Migration among Children 0-35 months, June 2018 and January 2019, n=2564

	**Child Age at First Migration, % (95% CI[*])**				
	**Born during Migration**	**0 to 5 months**	**6 to 11 months**	**≥ 12 months**	**P**
**All**	16.4 (15.0-17.8)	39.1 (37.2-40.9)	23.3 (21.6-24.9)	21.3 (19.7-22.8)	
**Origin**					
Bihar	19.8 (17.7-21.8)	42.3 (39.7-44.9)	21.4 (19.3-23.5)	16.6 (14.7-18.5)	
Jharkhand	12.9 (10.7-15.1)	35.8 (32.6-39.1)	25.3 (22.3-28.2)	26.0 (23.0-28.9)	
Other	11.7 (8.4-15.0)	34.4 (30.2-38.6)	25.7 (21.2-30.1)	28.3 (23.7-32.9)	<0.0001
**Caste**					
Scheduled Caste	19.1 (16.9-21.3)	40.2 (37.3-43.1)	22.6 (20.2-25.0)	18.1 (16.0-20.3)	
Scheduled Tribe	11.7 (9.2-14.3)	34.0 (30.2-37.7)	26.2 (22.8-29.6)	28.1 (24.7-31.5)	
Other Backward Class	17.7 (14.7-20.7)	44.0 (40.2-47.8)	20.7 (17.5-23.9)	17.7 (14.7-20.7)	
General Caste	11.5 (6.7-16.2)	33.1 (26.4-39.9)	25.5 (19.2-31.7)	29.9 (22.5-37.3)	<0.0001
**Parity of Mother**					
≤ 3	15.2 (13.6-16.9)	38.7 (36.5-41.0)	23.9 (21.9-25.9)	22.1 (20.1-24.1)	
≥ 4	18.9 (16.2-21.7)	39.8 (36.5-43.1)	21.8 (19.0-24.7)	19.4 (16.7-22.1)	0.053
**Mother's Education**					
No formal education	17.3 (15.7-18.8)	40.1 (38.0-42.1)	22.9 (21.1-24.6)	19.8 (18.1-21.5)	
Up to 8th standard	12.1 (8.2-15.9)	35.9 (30.1-41.7)	25.3 (20.1-30.5)	26.7 (21.6-31.9)	
Above 8th standard	10.5 (4.9-16.2)	28.1 (20.1-36.0)	25.4 (17.6-33.3)	36.0 (26.9-45)	<0.0001
**Household Size at Origin[†]**					
≤ 6	15.6 (13.9-17.3)	37.4 (35.1-39.8)	23.6 (21.5-25.7)	23.4 (21.4-25.5)	
≥ 7	17.8 (15.3-20.2)	41.7 (38.6-44.8)	22.7 (20.1-25.3)	17.8 (15.4-20.2)	0.003
**Wealth Quintile**					
Lowest	20.3 (16.8-23.9)	40.6 (36.5-44.8)	22.8 (19.2-26.4)	16.2 (13.1-19.4)	
Second	18.1 (14.8-21.4)	40.6 (36.3-44.8)	20.7 (17.2-24.2)	20.7 (17.2-24.1)	
Middle	17.9 (14.7-21.2)	39.4 (35.3-43.5)	21.4 (17.9-24.9)	21.2 (17.8-24.7)	
Fourth	15.2 (12.1-18.3)	37.2 (33.2-41.3)	27.1 (23.1-31.1)	20.5 (17.0-23.9)	
Highest	10.5 (7.9-13.2)	37.6 (33.5-41.8)	24.2 (20.4-27.9)	27.7 (23.8-31.5)	0.0001

[*] CI: confidence interval

[†] The definition of a household is a group of people who eat meals from the same kitchen
